# First-Episode Psychotic Patients Showed Longitudinal Brain Changes Using fMRI With an Emotional Auditory Paradigm

**DOI:** 10.3389/fpsyt.2020.593042

**Published:** 2020-12-11

**Authors:** Carlos González-Vivas, Gracián García-Martí, Pau Soldevila-Matías, Roberto Sanz-Requena, Eduardo J. Aguilar, María José Castro-Bleda, Luis Martí-Bonmatí, Julio Sanjuan

**Affiliations:** ^1^INCLIVA Health Research Institute, Clinical University Hospital, Valencia, Spain; ^2^Centro de Investigación Biomédica en Red de Salud Mental (CIBERSAM), Madrid, Spain; ^3^Radiology Department, Quirónsalud Hospital, Valencia, Spain; ^4^Department of Health Science, Valencia International University, Valencia, Spain; ^5^Department of Medicine, Valencia University, Valencia, Spain; ^6^Universitat Politècnica de València, Valencia, Spain

**Keywords:** first episode psychosis (FEP), fMRI—functional magnetic resonance imaging, longitudinal, emotional design model, paradigm, follow up

## Abstract

Most previous longitudinal studies of functional magnetic resonance imaging (fMRI) in first-episode psychosis (FEP) using cognitive paradigm task found an increased activation after antipsychotic medications. We designed an emotional auditory paradigm to explore brain activation during emotional and nonemotional word processing. This study aimed to analyze if longitudinal changes in brain fMRI BOLD activation is present in patients vs. healthy controls. A group of FEP patients (*n* = 34) received clinical assessment and had a fMRI scan at baseline and follow-up (average, 25-month interval). During the fMRI scan, both emotional and nonemotional words were presented as a block design. Results were compared with a pair of healthy control group (*n* = 13). Patients showed a decreased activation at follow-up fMRI in amygdala (*F* = 4.69; *p* = 0.04) and hippocampus (*F* = 5.03; *p* = 0.03) compared with controls. Middle frontal gyrus was the only area that showed a substantial increased activation in patients (*F* = 4.53; *p* = 0.04). A great heterogeneity in individual activation patterns was also found. These results support the relevance of the type of paradigm in neuroimaging for psychosis. This is, as far as we know, the first longitudinal study with an emotional auditory paradigm in FEP. Our results suggested that the amygdala and hippocampus play a key role in psychotic disease. More studies are needed to understand the heterogeneity of response at individual level.

## Introduction

Psychosis is a group of heterogeneous mental disorders characterized by important life disturbances, cognitive impairment, interpersonal problems, and, finally, high economic costs for health systems, including indirect costs such as unemployment and social support, and direct costs from hospitalizations during crises (1). First-episode psychosis (FEP) is defined as the first manifestation of a psychotic disorder meeting appropriate diagnostic symptom and time criteria (2), occurring in more than 3% of people at some point in life (3).

FEP has received a lot of interest from the scientific community in recent years, to increase recovery rates and prevent deterioration due to the emerging importance of an adequate and rapid response in the early stages of the disease (4–8). Prodromal factors have been investigated, conforming the clinical high-risk state for psychosis (CHR-P) knowledge based on two decades (9), seeking to clarify the most important risk factors that can predict the emerging of first symptom and answer why most high-risk individuals will never develop symptoms while being exposed to these risk factors, although meta-analyses report around 30% of high-risk individuals developing symptoms (1). In that sense, genetic and environmental interaction should also be considered, as they seem to work synergistically (6). In this sense, a great heterogeneity has been described within the CHR-P population, finding different subgroups prior to the emerging of FEP (10).

Despite this research effort, there are key remaining questions regarding FEP, the factors that explain the enormous clinical heterogeneity, the different course trajectories, and high relapse risks after improving pharmacological treatments and early interventions (6).

One main attempt to evaluate FEP is by using neuroimaging techniques. Most publications use MRI imaging measurements (11), ventricular dilation, and gray matter lost in chronic samples (12, 13), with similar results found in FEP patients (14). Treatment of naïve patients have shown gray matter deficits, mainly in the fronto-temporal, thalamo-cortical, and subcortical-limbic circuits (15, 16). There have been several attempts to link these structural findings with the outcomes and longitudinal trajectories of the disease, but clinical and methodological heterogeneity limits the conclusions (17).

Conversely, little is known about longitudinal changes in FEP during functional magnetic resonance imaging (fMRI) (18). One review, interested in the effects of medication on chronic patients, concluded that pharmacological antipsychotic treatments induce functional brain alterations in the frontal regions (19), but this conclusion has been reached by including different studies with an enormous methodological variance.

There are some problems with performing longitudinal fMRI studies: the cost of these techniques translates into low sample sizes across most studies (18, 19); the experimental dropouts in a difficult population to be recruited and evaluated; and the methodological heterogeneity during scans, which involves many different paradigms, usually visual or cognitive tasks (18). The type of task is a key issue in neuroimaging, since different areas of brain activation are shown as distinct task designs (such as emotional, cognitive, motor, attentional, verbal) and different sensory modalities (such as visual, auditory, resting state) are carried out. The distinctions between the strength of the MR scans are also noticeable, ranging from earlier studies with 1.5 T to later 7 T high field scanners, though their results are often intermixed.

Our group developed an emotional auditory paradigm based on some of the most common symptoms reported by FEP patients (20, 21). Some interesting results have been found using this method in chronic samples (22), but the issue of longitudinal activation changes in FEP remains unclear.

There are relatively few publications studying longitudinal activation changes in FEP cohorts. We recently conducted what seems to be the only systematic review about this question and gathered the results of 13 selected studies (18). First of all, there was a great methodological heterogeneity across the included studies, involving different scan powers, different intervals between scans, different regions of interest, and different tasks and modalities. Taken together the activation and connectivity studies, two main comparisons were carried out. First, the patient's group was compared with the healthy control's group in basal assessment, finding multiple studies with hypoactivation in several brain areas in the patients' group. The most frequently hypoactivated area was the prefrontal cortex, followed by limbic structures, occipital cortex, basal ganglia, thalamus, hippocampus, temporal cortex, and parietal cortex. The number of studies reporting hyperactivation in patients vs. controls was marginal. This was in contrast to the results of a previous selective review that concluded a hippocampal and subcortical hyperactivation pattern in naive patients (15).

Secondly, we compared the results of the second MR scans within the patient's group with respect to the basal assessment, with a general increased activation (which we called “normalization”) in the vast majority of included studies, especially in prefrontal cortex, basal ganglia, parietal cortex, and occipital cortex. These results were explained in the reference studies as treatment effects (18).

Under our emotional auditory paradigm, an important role of amygdala in emotional processing in chronic patients with hallucinations can be observed (20). We hypothesize that evaluating a FEP with our emotional paradigm in fMRI will probably show different activation patterns, especially in amygdala, between patient and healthy control groups. We also expect that the activation will decrease in those regions after treatment. The purpose of this study is to compare the longitudinal activation results evaluated with an emotional auditory paradigm during fMRI scans in a FEP patient with a heathy control group. The potential of this method as a biomarker for FEP will be discussed.

## Materials and Methods

### Participants

Patients were recruited from the outpatient psychiatry clinics. People from area 5 of Valencia, who experienced psychotic symptoms for the first time in life were submitted to the Valencia Clinic Hospital First-Episode Unit for evaluation and treatment. Control subjects were paired by age and educational level. All subjects, patients and controls, were older than 18 years old and provided written informed consent according to the Local Ethical Committee.

There were 35 adult patients with a first F20–F29 diagnosis. Diagnoses were based on the Structured Clinical Interview for DSM-IV Axis I Disorders. Two weeks after their diagnosis, all the patients underwent a semistructured interview including sociological information and clinical scales. After the clinical evaluation, they were proposed to be scanned with two fMRI scans, with a minimum interval of 6 months between them.

Healthy controls were volunteers without financial remuneration recruited from the Valencia Clinic Hospital and Valencia University staff. They were selected after a mini personal interview to exclude people with the following: (a) personal or family clinical records of psychiatric illness; (b) people who suffered from a medical illness that potentially affected cerebral function; and (c) people who met present or past criteria for psychoactive drug abuse or dependence (except for tobacco). Sociodemographic data is gathered in Table 1.

**Table 1:** Sociodemographical characteristics of subjects.

	**FEP patients group**	**Healthy controls group**
	**(*n* = 35)**	**(*n* = 13)**
Age (years at first scan)	29.92 ± 8.90	35.70 ± 10.74
Gender (male/female)	28/6	8/5
Education level	Illiteracy = 1	Illiteracy = 0
	Primary = 9	Primary = 1
	Secondary = 6	Secondary = 2
	High school = 9	High school = 4
	University = 10	University = 6
Work status	Active = 5	Active = 9
	Unemployed = 18	Unemployed = 2
	Disabled = 1	Disabled = 0
	Student = 11	Student = 2
Coexistence	Alone = 1	Alone = 4
	Origin family = 28	Origin family = 3
	Own family = 2	Own family = 4
	Other = 4	Other = 2
Interval (months between scans)	25.60 ± 19.50	19.68 ± 13.03

### Clinical Measurements

Patients were assessed by a personnel trained in FEP evaluation (JS). Interviews were conducted during the first episode and a few days after the start of treatment. All patients were assessed at least twice, coinciding with the time difference between scans. Pharmacological options were registered, even if depot administration was implemented, and others are combinations with non-antipsychotic medications.

The Clinical Global Impressions scale (CGI) and the Global Assessment of Functioning scale (GAF) were taken into account and complemented by the Positive and Negative Syndrome scale (PANSS). The results of clinical measurements in baseline and follow-up are summarized in Table 2.

**Table 2:** Clinical characteristics of subjects.

	**Basal (*n* = 35)**	**Follow-up (*n* = 35)**	***p*-values**
CGI	4.23 ± 0.770	3.49 ± 1.040	0.002
GAF	62.20 ± 11.045	67.71 ± 15.007	0.040
PANSS: T	59.69 ± 13.809	47.17 ± 16.224	0.000
PANSS: P	15.06 ± 5.525	11.17 ± 4.914	0.002
PANSS: N	13.71 ± 4.430	11.34 ± 3.925	0.003
PANSS: PG	30.06 ± 6.708	24.17 ± 8.607	0.002
Medication	1st G APS = 35 (paliperidone = 12, olanzapine = 11, aripiprazole = 5, risperidone = 3, amisulpride = 2, quetiapine = 2)		
	APS combination = 9		
	Anticholinergics = 2		
	Benzodiazepines = 26		
	Antidepressants = 4		
	Mood stabilizers = 3		
	Mean CPZ equivalent dose: 266 mg. SD = 1.82		

### fMRI Emotional Paradigm

An emotional auditory fMRI stimulation paradigm, designed by our group (20, 21), was employed in this study, consisting of 13 emotional Spanish words taking into account their frequency of presentation in the patient's hallucinations, and 13 neutral words with a similar number of syllables from a database (23). A set of earphones, connected by a pair of air tubes to an external audio player, was used. Two different fMRI acquisitions, neutral and emotional, were conducted for each subject. The neutral words were presented first. The paradigm used a block design approach to present the words. Four blocks of rest and four blocks of stimulation were interleaved and presented to each participant. Each block lasted 20 s. Subjects were informed before the MR acquisition about the experiment and were asked to focus their attention to the words.

### MR Scanning Protocol

fMRI scans were obtained at baseline and follow-up with a 3-T magnet (Achieva, Philips Medical Systems, Best, The Netherlands) and a multitransmit parallel transmission technology. Brain fMRI images were obtained using a 32-channel head coil and a dynamic echo planar imaging (EPI) T2^*^-weighted sequence (repetition time = 2,000 ms; echo time = 30 ms; flip angle 90; slice thickness = 3.50 mm with no interslice gap; acquisition matrix = 128 × 128; voxel size = 1.80 × 1.80 × 3.50 mm), covering the whole brain with 40 contiguous slices. The total fMRI acquisition duration was 160 s.

After acquisition, images were qualitatively reviewed by a radiologist and a computer engineer, to ensure data quality. Data was then anonymized and transferred to a workstation for postprocessing analysis.

### Analyses

Statistical Parametric Mapping version 12 software (SPM, The Wellcome Center for Human Neuroimaging, London, UK) and MATLAB R2015a (The MathWorks, Natick, MA, USA) were used to process the fMRI series. First, images were spatially realigned to minimize potential movements during the MR acquisition. Motion maps were generated for each fMRI series and analyzed to discard those subjects with translation movements (x, y, z) >1.80 mm (voxel size). No subjects were discarded after this step. Secondly, a slice timing correction was used to adjust for temporal delays between the first and the last slices in each fMRI dynamic. Then, a spatial normalization algorithm was applied to normalize data with respect to a standard template (MNI350, Montreal Neurological Institute). Finally, the normalized images were smoothed by a 6-mm FWHM Gaussian smoothing kernel.

A first-level analysis was conducted for each individual subject. In order to increase specificity, fMRI response was measured during emotional words in all subjects in areas previously reported as significant with the emotional paradigm, including amygdala, hippocampus, cingulated gyrus, insula, frontal, and temporal regions (21, 22, 24, 25). In these areas, a relative measurement of functional activation [percent signal change (PSC)] was calculated as the proportional signal difference between the signal averaged in the rest blocks and the signal averaged in the stimulation period was measured. To minimize the potential effect of spurious values, a robust strategy was used to obtain the mean and median values for each region of interest (ROI), discarding those values with two time differences, above or below the standard deviation of the PSC in each area. For each ROI and case, the PSC was computed voxel by voxel. To minimize the bias associated to multiple statistical comparisons and to control the expected number of false positives, a Bonferroni correction was applied at voxel level through a family-wise error (FWE) rate correction.

A full-factorial ANOVA model was constructed and evaluated to assess for changes in fMRI activation between subjects by accounting for two main factors: clinical group (patients vs. controls) and longitudinal acquisition (basal—MRI1 and follow-up—MRI2).

## Results

### fMRI Response to Emotional Stimulation

**Figure 1:**
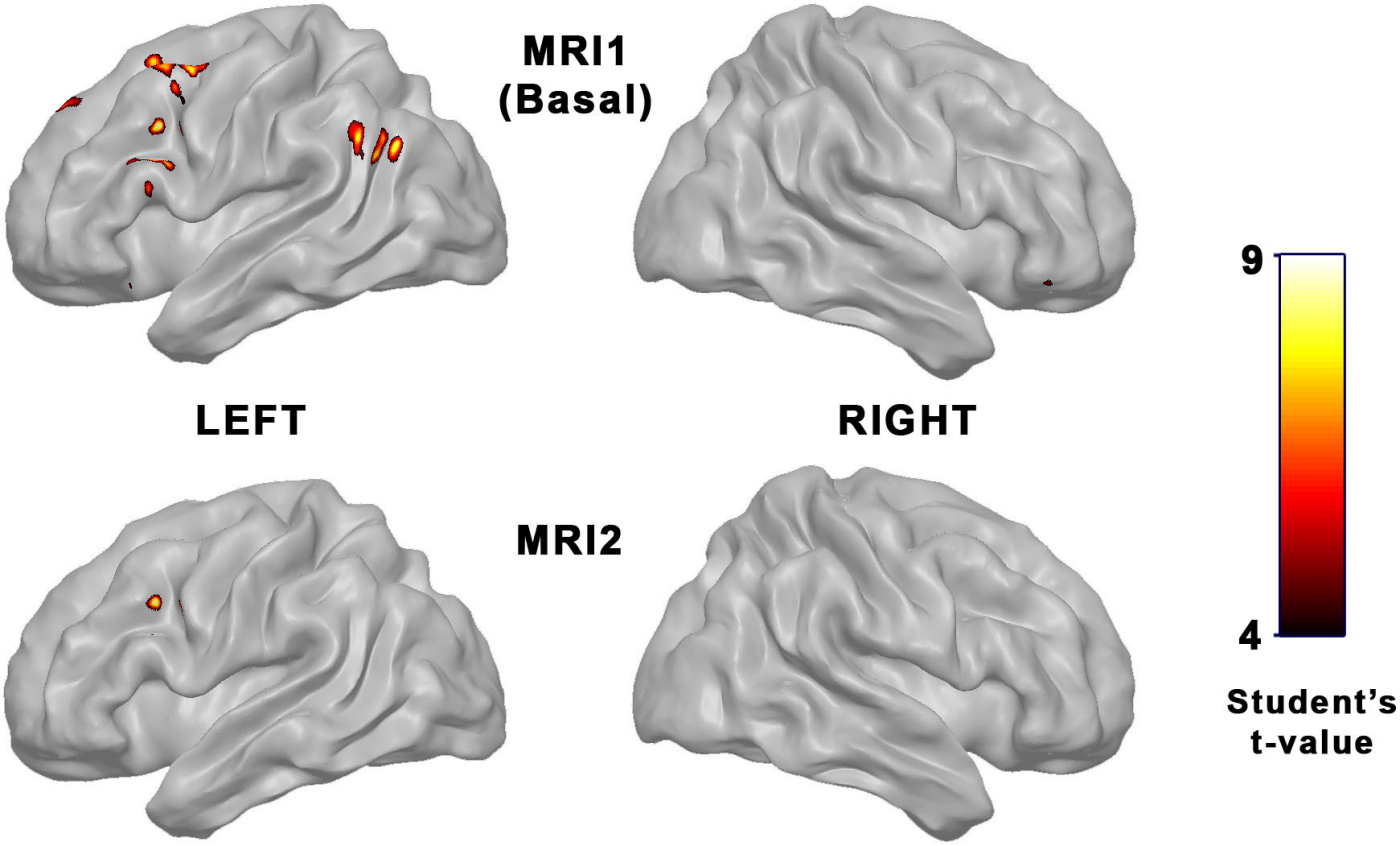
Parametric maps showing areas of hyperactivation in FEP patients vs. controls when emotional stimulation was applied. **Top row:** Differences in basal fMRI evaluation. **Bottom row:** Differences in follow-up fMRI evaluation (*p* < 0.05 FWE-corrected).

Compared with the control subjects, FEP patients showed hyperactivated areas when the emotional stimulation was applied. In the first basal fMRI acquisition, the hyperactivated areas were located mainly at frontal and temporal regions. The follow-up fMRI examination showed lesser differences between FEP and healthy controls, with only a small portion of the middle frontal lobe being hyperactivated (see Table 3 and Figure 1). A complete list of areas, figures, and tables showing the whole-brain full-activation maps for each group is available as Tables S1, S2 and Figures S1, S2.

**Table 3:** Areas of hyperactivation in FEP patients vs. controls when emotional stimulation was applied.

**MRI1 (basal)**				**MRI2 (follow-up)**			
***T* Student**	**Coordinates**	**Label**	**Brodmann**	***T* Student**	**Coordinates**	**Label**	**Brodmann**
9.22	[−28, 16, 58]	Frontal_Mid_L	08	7.30	[−38, 40, 34]	Frontal_Mid_L	46
9.02	[−12, 46, 44]	Frontal_Sup_Medial_L	09				
8.92	[−46, 8, 34]	Frontal_Inf_Oper_L	48				
8.84	[−32, 16, 34]	Frontal_Inf_Oper_L	44				
8.72	[−38, 16, −24]	Temporal_Pole_Sup_L	38				
8.49	[48, 34, −16]	Frontal_Inf_Orb_R	47				

*Left MRI1 (basal) and right MRI2 (follow-up) (p < 0.05 FWE-corrected)*.

### Effect Over Time

**Figure 2:**
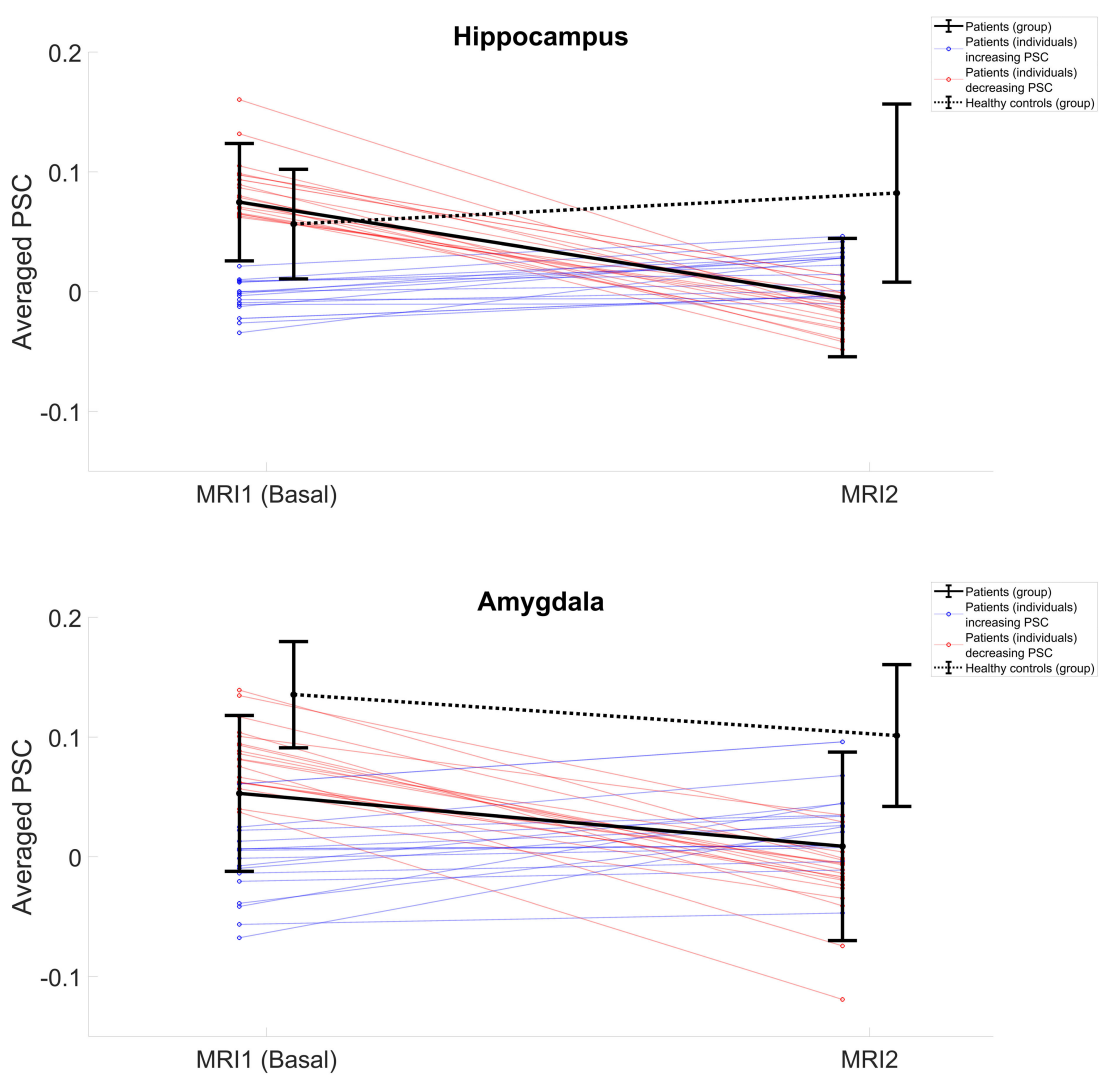
PSC changes over time, from basal to follow-up fMRI examination. **Top row:** PSC in hippocampus. **Bottom row:** Values in amygdala. Black lines represent the averaged group values for FEP patients (continuous line) and healthy controls (dotted line). Colored lines represent the individual curves for patients who increment PSC over time (blue) and those who showed a longitudinal PSC decrement (red).

FEP patients showed a significant reduction in fMRI activation in the follow-up MRI examination of the hippocampus (*F* = 4.81; *p* = 0.03). Other areas including amygdala, cingulated gyrus, insula, frontal, and temporal regions did not show functional changes over time. Likewise, no significant differences were found in healthy control subjects in these areas between both fMRI examinations (see Figure 2 and Table 4).

**Table 4:** ANOVA showing functional PSC values of FEP patients (left) and healthy controls (right) for both, basal and follow-up fMRI evaluations.

	**FEP patients**				**Healthy controls**			
	**PSC**		***F***	***P*-value**	**PSC**		***F***	***p*-value**
	**Basal (MRI1)**	**Follow-up (MRI2)**			**Basal (MRI1)**	**Follow-up (MRI2)**		
Amygdala	7.82 (11.34)	2.70 (13.90)	1.58	0.21	12.09 (8.80)	11.75 (11.36)	0.33	0.57
Hippocampus	8.64 (8.30)	−1.01 (8.79)	4.81	0.03^*^	6.65 (9.57)	9.83 (13.67)	0.86	0.36
Cingulated Gyrus	5.15 (19.64)	0.15 (30.15)	0.69	0.41	−0.65 (17.82)	3.28 (20.69)	0.27	0.61
Frontal	2.06 (9.48)	3.51 (10.53)	0.06	0.80	−2.40 (9.47)	0.65 (10.41)	0.28	0.60
Temporal	7.89 (7.60)	4.66 (20.79)	0.01	0.93	5.05 (7.11)	14.23 (15.56)	0.95	0.34
Insula	−1.30 (19.85)	0.69 (24.85)	0.14	0.71	3.12 (12.03)	3.14 (16.84)	0.00	1.00

* *p < 0.05*.

Although averaged-group values in the hippocampus showed statistically significant changes over time, a marked heterogeneity in the behavior of the curves was detected when observing particular subjects. While some patients showed a clear increase of PSC activation during follow-up fMRI evaluations, others exhibited a marked reduction over time (Figure 2).

### Effect Over Clinical Groups

At baseline fMRI, FEP patients showed a significant reduction of activation compared with controls in the amygdala (*F* = 4.67; *p* = 0.04). By contrast temporal (*F* = 3.96; *p* = 0.04) and frontal (*F* = 4.23; *p* = 0.04) regions were hyperactivated in FEP patients. Other areas including the hippocampus, cingulated gyrus, and insula did not show significant differences between groups.

In the follow-up study, both amygdala (*F* = 4.69; *p* = 0.04) and hippocampus (*F* = 5.03; *p* = 0.03) showed a significant fMRI reduction in patients vs. controls while the middle frontal gyrus (*F* = 4.53; *p* = 0.04) showed a hyperactivation in patients (see Figure 2 and Table 5).

**Table 5:** ANOVA showing functional PSC values at basal (left) and follow-up (right) evaluation for FEP patients and healthy control subjects.

	**Basal (MRI1)**				**Follow-up (MRI2)**			
	**PSC**		***F***	***p*-value**	**PSC**		***F***	***p*-value**
	**FEP patients**	**Healthy controls**			**FEP patients**	**Healthy controls**		
Amygdala	7.82 (11.34)	12.09 (8.80)	4.67	0.04^*^	2.70 (13.90)	11.75 (11.36)	4.69	0.04^*^
Hippocampus	8.64 (8.30)	6.65 (9.57)	0.22	0.65	−1.01 (8.79)	9.83 (13.67)	5.03	0.03^*^
Cingulated gyrus	5.15 (19.64)	−0.65 (17.82)	0.87	0.36	0.15 (30.15)	3.28 (20.69)	0.12	0.73
Frontal	2.06 (9.48)	−2.40 (9.47)	4.23	0.04^*^	3.51 (10.53)	0.65 (10.41)	4.53	0.04^*^
Temporal	7.89 (7.60)	5.05 (7.11)	3.96	0.04^*^	4.66 (20.79)	14.23 (15.56)	2.27	0.14
Insula	−1.30 (19.85)	3.12 (12.03)	0.57	0.46	0.69 (24.85)	3.14 (16.84)	0.11	0.74

* *p < 0.05*.

An analysis of a possible interaction between clinical symptoms and longitudinal changes in fMRI was made through PANSS result data: general PANSS score, positive subscale, negative subscale, and general psychopathology subscale. No significant correlation between these scores and longitudinal fMRI changes were found (Table 6).

**Table 6:** Correlations between differences in activation in amygdala and hippocampus and clinical variables (PANSS subscales measurements and pharmacological treatment in chlorpromazine equivalent doses) measured as longitudinal percentage change.

	**DIFAMIG**	**DIFHIPOC**	**PC_P**	**PC_N**	**PC_G**
**DIFAMIG**
Pearson correlation	1	0.717^**^	−0.044	0.000	0.092
Sig. (2-tailed)		0.000	0.800	0.998	0.598
*N*	35	35	35	35	35
**DIFHIPOC**					
Pearson correlation	0.717^**^	1	−0.132	−0.041	0.117
Sig. (2-tailed)	0.000		0.451	0.813	0.503
*N*	35	35	35	35	35
**PANSS_P**					
Pearson correlation	−0.044	−0.132	1	0.959^**^	0.223
Sig. (2-tailed)	0.800	0.451		0.000	0.197
*N*	35	35	35	35	35
**PANSS_N**					
Pearson correlation	0.000	−0.041	0.959^**^	1	0.372^*^
Sig. (2-tailed)	0.998	0.813	0.000		0.028
*N*	35	35	35	35	35
**PANSS_G**					
Pearson correlation	0.092	0.117	0.223	0.372^*^	1
Sig. (2-tailed)	0.598	0.503	0.197	0.028	
*N*	35	35	35	35	35
**CPZ_EQ**					
Pearson correlation	−0.082	−0.041	−0.067	−0.131	−0.045
Sig. (2-tailed)	0.638	0.815	0.704	0.453	0.799
*N*	35	35	35	35	35

* *Correlation is significant at the 0.05 level (2-tailed)*.

** *Correlation is significant at the 0.01 level (2-tailed)*.

In order to look at the possible antipsychotic effect on fMRI, chlorpromazine-equivalent doses were calculated and registered (Table 2). The relationship between longitudinal activation change and clinical variables has been calculated, showing that there is no correlation between longitudinal fMRI changes and antipsychotic dose (Table 6).

## Discussion

This first longitudinal study investigating fMRI changes in FEP using an emotional auditory paradigm reveals an increased activation at baseline that is reduced in follow-up. Interestingly, in our previous systematic review of fMRI longitudinal studies in FEP, the most common finding was just the opposite. Patients with FEP showed at baseline a hypoactivation mainly in PFC, basal ganglia, limbic system, hippocampus, and the ACC, which was increased (“normalized”) after antipsychotic treatment during follow-up (18).

The most likely explanation for understanding the differences between our results and previous studies is the fMRI paradigm. Most previous fMRI longitudinal studies in FEP used resting states or different paradigms that included cognitive tasks. The main problem of using any kind of cognitive task in psychotic patients is the attentional bias as attentional deficits are one of the most common findings in FEP patients (26). In fMRI studies, these deficits correlate with general hypoactivation mainly in the PFC (27). Moreover, hypoactivation in the PFC has been mentioned classically as one of the most consistent findings in chronic schizophrenia (28). However, the type of paradigm we used in this study does not require a focus of attention or cognitive performance but rather reflects the brain's response to direct emotional stimulation. In support of this, a hyperactivation of the amygdala at baseline that decreased after treatment with cognitive therapy was observed in schizophrenia patients with persistent hallucinations (22).

The fact that amygdala and hippocampus showed the most significant changes deserves an explanation. First, both areas were the most clearly affected in our previous studies using the same paradigm in chronic patients (29). Secondly, these areas are implicated in the emotional response and memory, fitting with the Kapur's model in which the core of psychosis could be an abnormal “salience” to external stimuli (30) or, as more recently suggested, an abnormal connectivity between perceptual, emotional, and memory brain areas (31).

Quite relevant is the fact that everything mentioned so far relates with mean group variations in brain activation. It is important to consider the great individual heterogeneity in the results. This heterogeneity is not only in the baseline but also in the path of brain activation changes. Some patients show a decrease in brain activation during follow-up, while another group presents just the opposite (Figure 2). There are several possible explanations for these results. The simplest one is that the heterogeneity in brain activation is due to the clinical heterogeneity of the population. Our study sample was based on patients with first episodes of psychosis. The first psychotic episodes can evolve very favorably (brief reactive psychosis) or tend toward chronicity either in a continuous deterioration or in an episodic way (32). However, we did not find a clinical difference at baseline with regard to the pattern of longitudinal activation changes (see Figures S1, S2 and Tables S1, S2). That said, it is possible to speculate that differences in brain activation are directly related with the clinical state at the moment of fMRI. So, the same patient could have a different degree of activation, depending on the moment of fMRI acquisition. Moreover, it is important to consider medication and psychosocial interventions as important confounding variables, and it is worth mentioning that patients were treated under naturalistic conditions with individualized therapeutic approaches. The results of this study showed the relevance of making an analysis at an individual base instead of just comparing the mean group.

This study has some limitations. Although most of the fMRI studies involve <30 patients, it remains limiting for the field, and this study must be interpreted wisely due to the relatively small sample size recruited. Differences between sample sizes in the HC and FEP groups and gender distribution should also be taken into account. The second limitation is about the different intervals between scans, particularly in the case of the FEP group. As we are comparing brain function after symptom's onset, the natural effects of neurodevelopment should be controlled by adjusting the time between scans. Thirdly, although we did not find a significant correlation between clinical symptoms and brain activation (Table 6) in our sample, like all FEP samples, there is a great clinical heterogeneity that it cannot be ignored. Different types of psychosis are included, which probably involve different disease phenotypes and, as we have shown in this paper, different pathways in brain function. Finally, according to this clinical heterogeneity, a wide range of pharmacological treatments appear in our sample. Although we did not find a significant relationship of the antipsychotic equivalent dose and fMRI activation, it cannot be ruled out that different combinations of medications in longitudinal studies may introduce changes in natural brain function. More analyses are needed, studying individual cases and possible patient subgroups [including genetic data (33)] to improve our knowledge of brain function and the course of psychosis.

Conversely, this study has some strengths that should be listed. The main strength is that this is the first longitudinal study investigating longitudinal fMRI changes in FEP samples with an emotional auditory paradigm. Paradigm election is a key question in neuroimaging, and there is a lack of longitudinal studies using noncognitive task during scans. Another positive point of this study is to incorporate clinical assessments in addition to neuroimage acquisition data. Neuroimaging and clinical data may be gathered to study disease course variables in subsequent analyses.

In conclusion, we have shown that brain activation under an emotional auditory paradigm during fMRI scans change longitudinally in FEP patients. A significant decrease in activation in the amygdala and hippocampus is observed in patients compared with healthy subjects. Still, we have also found a great clinical and activation heterogeneity, which may reflect different pathways of the disease.

## Data Availability Statement

The datasets presented in this article are not readily available because of ethical restrictions. Reasonable requests to access the datasets should be directed to Julio Sanjuan, julio.sanjuan@uv.es.

## Ethics Statement

The studies involving human participants were reviewed and approved by INCLIVA Ethical Committee. The patients/participants provided their written informed consent to participate in this study.

## Author Contributions

JS, CG-V, GG-M, LM-B, and EA designed the study. JS obtained the funding and supervised the study. GG-M made the postprocessing of fMRI data. PS-M made the clinical evaluation. RS-R made the imaging acquisition. MC-B contributed to the data analysis. CG-V wrote the first draft of the article. JS, CG-V, and GG-M wrote the final version. All authors contributed to the article and approved the submitted version.

## Conflict of Interest

The authors declare that the research was conducted in the absence of any commercial or financial relationships that could be construed as a potential conflict of interest.
