# Supplementary material for: First-Episode Psychotic Patients Showed Longitudinal Brain Changes Using fMRI With an Emotional Auditory Paradigm
Source: Front Psychiatry. 2020 Dec 11;11:593042. doi: 10.3389/fpsyt.2020.593042 (PMC7794005; doi:10.3389/fpsyt.2020.593042)
Supplement: Supplementary file 1 [file Table_1.docx]

**Table S1**. Areas of emotional functional activation in FEP patients in basal (MR1) and follow-up (MRI2) fMRI evaluation (p<0.05 FWE-corrected).

| **MRI1 (Basal)** | | | |  | **MRI2 (Follow-up)** | | | |
| --- | --- | --- | --- | --- | --- | --- | --- | --- |
|  |  |  |  |  |  |  |  |  |
| T Student | Coordinates | Label | Brodmann |  | T Student | Coordinates | Label | Brodmann |
| 12.87 | [-64 -22 -2] | Temporal_Sup_L | 48 |  | 11.14 | [-64 -22 -2] | Temporal_Sup_L | 21 |
| 12.27 | [62 -14 -8] | Temporal_Mid_R | 21 |  | 10.71 | [64 -24 -2] | Temporal_Mid_R | 21 |
| 6.25 | [52 26 18] | Frontal_Inf_Tri_R | 48 |  | 7.54 | [-48 20 22] | Frontal_Inf_Oper_L | 48 |
| 4.90 | [-52 -40 26] | SupraMarginal_L | 48 |  | 5.97 | [54 16 18] | Frontal_Inf_Tri_R | 48 |
| 4.87 | [22 -4 -22] | Hippocampus_R | 28 |  | 5.48 | [52 26 -6] | Frontal_Inf_Orb_R | 47 |
| 4.59 | [64 -38 26] | SupraMarginal_R | 48 |  | 5.30 | [-25 1 -19] | Amygdala_L | 34 |
| 4.39 | [-50 -52 -22] | Temporal_Inf_L | 37 |  | 5.10 | [-6 32 48] | Frontal_Sup_Medial_L | 08 |
| 4.32 | [-8 10 -2] | Caudate_L | 25 |  | 5.01 | [62 -38 24] | SupraMarginal_R | 48 |
| 4.17 | [22 -1 -19] | Amygdala_R | 34 |  | 4.83 | [26 8 -28] | Temporal_Pole_Sup_R | 28 |
