# Supplementary material for: First-Episode Psychotic Patients Showed Longitudinal Brain Changes Using fMRI With an Emotional Auditory Paradigm
Source: Front Psychiatry. 2020 Dec 11;11:593042. doi: 10.3389/fpsyt.2020.593042 (PMC7794005; doi:10.3389/fpsyt.2020.593042)
Supplement: Supplementary file 2 [file Table_2.docx]

**Table S2.** Areas of emotional functional activation in healthy control subjects in basal (MR1) and follow-up (MRI2) fMRI evaluation (p<0.05 FWE-corrected).

| **MRI1 (Basal)** | | | |  | **MRI2 (Follow-up)** | | | |
| --- | --- | --- | --- | --- | --- | --- | --- | --- |
|  |  |  |  |  |  |  |  |  |
| T Student | Coordinates | Label | Brodmann |  | T Student | Coordinates | Label | Brodmann |
| 8.90 | [-64 -32 8] | Temporal_Mid_L | 21 |  | 7.69 | [-66 -28 -4] | Temporal_Mid_L | 21 |
| 8.48 | [62 -12 -10] | Temporal_Mid_R | 21 |  | 6.70 | [62 -12 -10] | Temporal_Mid_R | 21 |
| 5.76 | [52 2 50] | Precentral_R | 06 |  | 6.66 | [-40 26 0] | Insula_L | 47 |
| 5.89 | [-20 -8 -20] | ParaHippocampal_L | 35 |  | 6.45 | [52 4 50] | Precentral_R | 06 |
| 4.81 | [28 -66 -26] | Cerebelum_6_R | 19 |  | 6.01 | [46 22 26] | Frontal_Inf_Tri_R | 48 |
| 4.42 | [-40 -6 40] | Precentral_L | 06 |  | 5.96 | [52 30 -2] | Frontal_Inf_Orb_R | 45 |
| 4.24 | [-52 -42 26] | SupraMarginal_L | 48 |  | 4.92 | [10 2 66] | Supp_Motor_Area_R | 06 |
| 4.19 | [4 31 -7] | Cingulum_Ant_R | 11 |  | 4.90 | [-28 -16 -14] | Hippocampus_L | 20 |
|  |  |  |  |  | 4.74 | [-54 18 12] | Frontal_Inf_Oper_L | 48 |
