# Supplementary figures and images for: First-Episode Psychotic Patients Showed Longitudinal Brain Changes Using fMRI With an Emotional Auditory Paradigm
Source: Front Psychiatry. 2020 Dec 11;11:593042. doi: 10.3389/fpsyt.2020.593042 (PMC7794005; doi:10.3389/fpsyt.2020.593042)

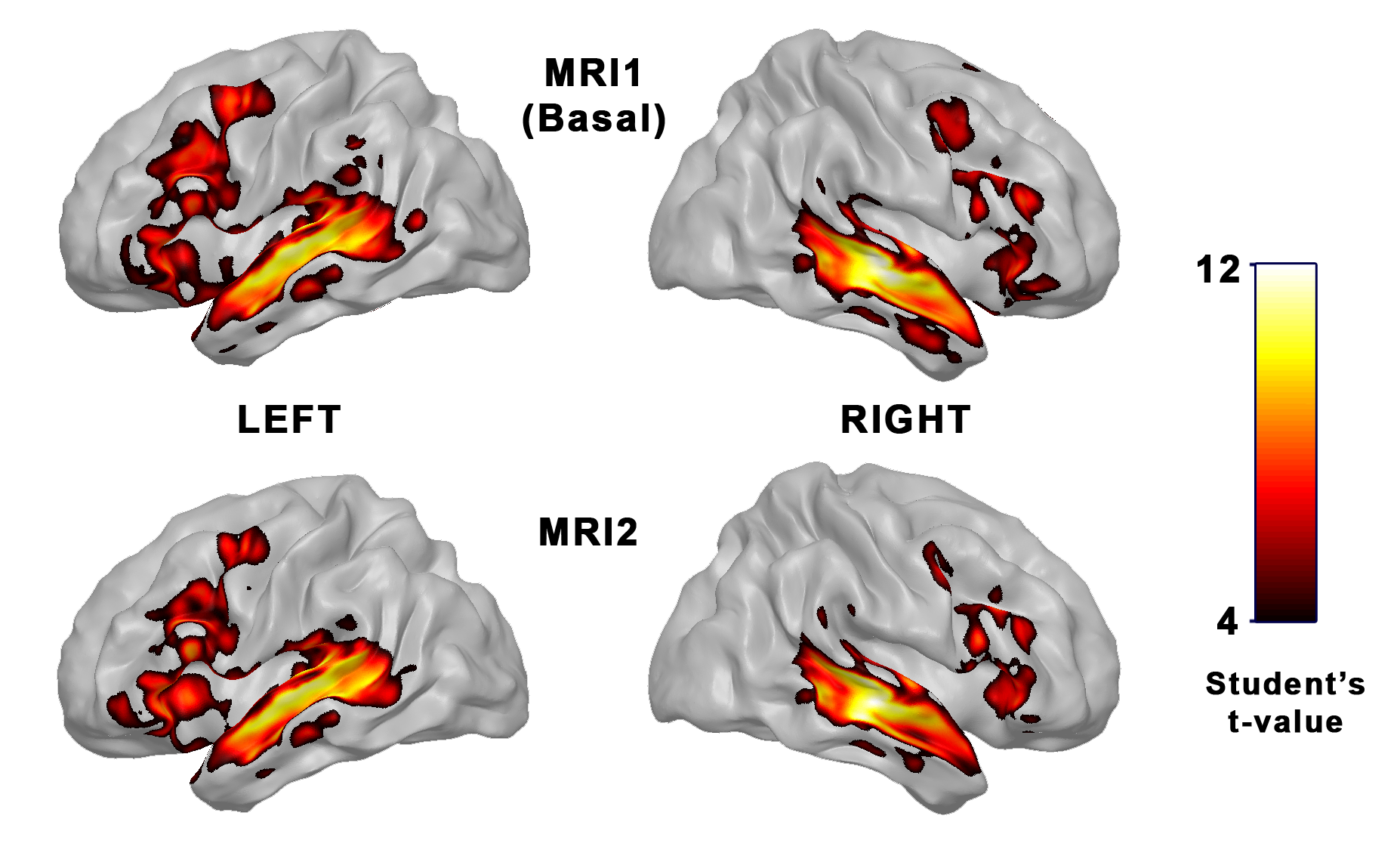

Supplement: Supplementary file 3 [file Image_1.tif]

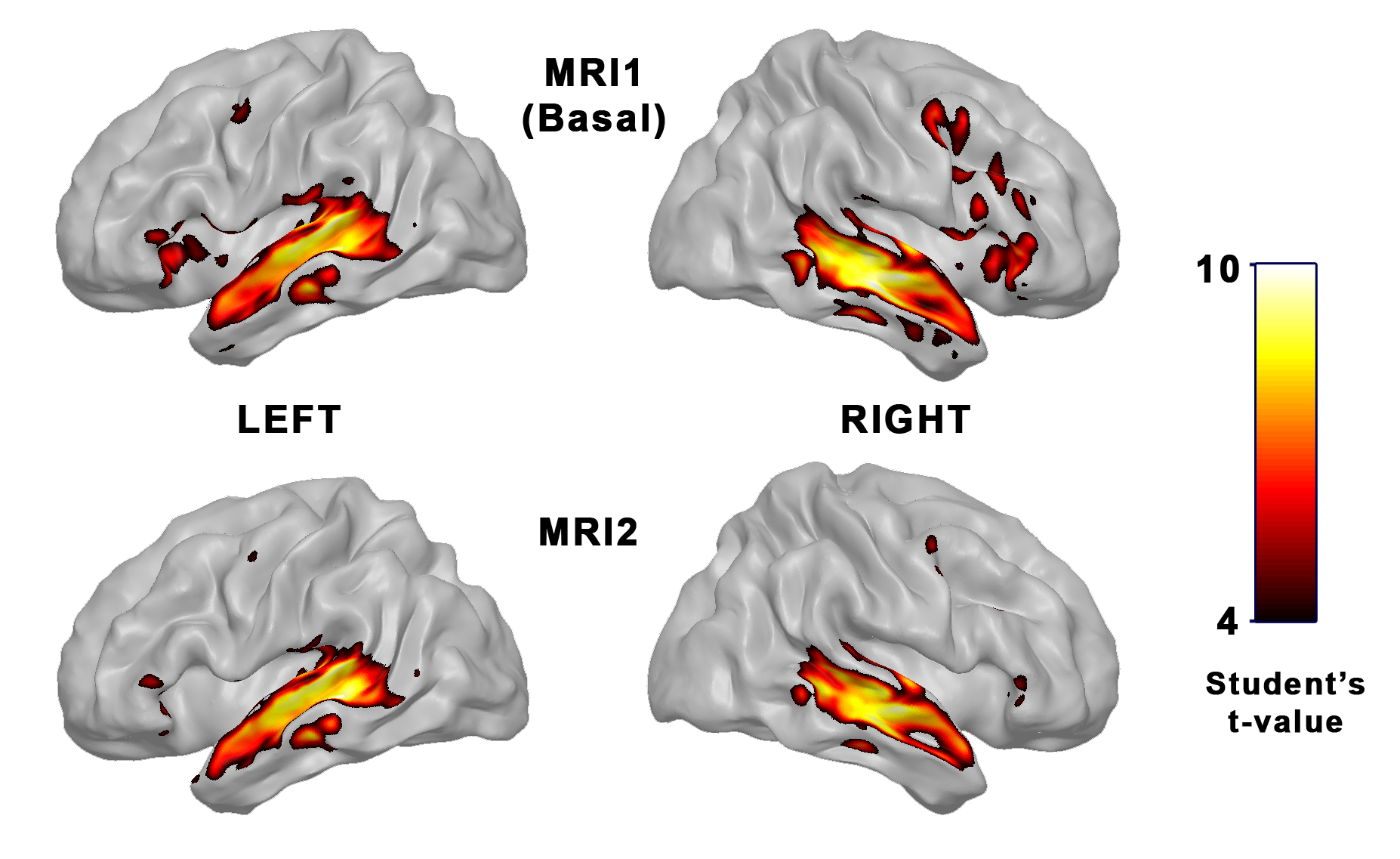

Supplement: Supplementary file 4 [file Image_2.tif]
